# CD206^+^CD68^+^ mono-macrophages and serum soluble CD206 level are increased in antineutrophil cytoplasmic antibodies associated glomerulonephritis

**DOI:** 10.1186/s12865-022-00529-w

**Published:** 2022-11-15

**Authors:** Xiao-Ning An, Zhao-Nan Wei, Yin-Yin Xie, Jing Xu, Yan Shen, Li-Yan Ni, Hao Shi, Ping-Yan Shen, Wen Zhang, Yong-Xi Chen

**Affiliations:** 1 Department of Nephrology, Ruijin Hospital Affiliated to Shanghai Jiaotong University, School of Medicine, No. 197, Ruijin Er Rd, Shanghai, 200025 People’s Republic of China; 2 Department of Nephrology, Xinrui Hospital, Ruijin Hospital Wuxi Branch, Wuxi, Jiangsu, People’s Republic of China; 3 Shanghai Institute of Hematology, State Key Laboratory of Medical Genomics, National Research Center for Translational Medicine at Shanghai, Ruijin Hospital Affiliated to Shanghai Jiaotong University School of Medicine, Shanghai, People’s Republic of China; 4 Research Center for Experimental Medicine, Ruijin Hospital Affiliated to Shanghai Jiaotong University, School of Medicine, Shanghai, People’s Republic of China; 5 Department of Nephrology, No. Six People’s Hospital Affiliated to Shanghai Jiaotong University, Shanghai, People’s Republic of China

**Keywords:** Antineutrophil cytoplasmic antibodies, Glomerulonephritis, CD206, Mono-macrophage, ANCA

## Abstract

**Background:**

Antineutrophil Cytoplasmic Antibodies (ANCA) associated glomerulonephritis (AGN) is a group of autoimmune diseases and mono-macrophages are involved in its glomerular injuries. In this study, we aim to investigate the role of CD206^+^ mono-macrophages in AGN.

**Methods:**

27 AGN patients (14 active AGN, 13 remissive AGN) together with healthy controls (n = 9), disease controls (n = 6) and kidney function adjusted controls (n = 9) from Department of Nephrology, Ruijin hospital were recruited. Flow cytometry was used to study proportion of CD206^+^ cells in peripheral blood. Immunohistochemistry for CD206 staining was performed and CD206 expression was scored in different kidney regions. Serum soluble CD206 (sCD206) was measured by enzyme-linked immunosorbent assay (ELISA). We also generated murine myeloperoxidase (MPO) (muMPO) ANCA by immunizing *Mpo*^−/−^ mice. Mouse bone marrow-derived macrophages (BMDMs) from wild C57BL/6 mice and peripheral blood mononuclear cell (PBMC) derived macrophages from healthy donors were treated with MPO ANCA with or without its inhibitor AZD5904 to investigate the effects of MPO-ANCA on CD206 expression.

**Results:**

The proportion of peripheral CD206^+^CD68^+^ cells in active AGN patients were significantly higher than that in remissive patients (*p* < 0.001), healthy controls (*p* < 0.001) and kidney function adjusted controls (*p* < 0.001). Serum sCD206 level in active AGN patients was higher than that in healthy controls (*p* < 0.05) and remissive patients (*p* < 0.01). Immunohistochemistry showed CD206 was highly expressed in different kidney regions including fibrinoid necrosis or crescent formation, glomeruli, periglomerular and tubulointerstitial compartment in active AGN patients in comparison with disease controls. Further studies showed MPO ANCA could induce CD206 expression in BMDMs and PBMC derived macrophages and such effects could be reversed by its inhibitor AZD5904.

**Conclusion:**

ANCA could induce CD206 expression on mono-macrophages and CD206^+^ mono-macrophages are activated in AGN. CD206 might be involved in the pathogenesis of AAV and may be a potential target for the disease.

**Supplementary Information:**

The online version contains supplementary material available at 10.1186/s12865-022-00529-w.

## Introduction

Antineutrophil Cytoplasmic Antibodies (ANCA) associated glomerulonephritis (AGN) is a group of autoimmune diseases which include microscopic polyangiitis (MPA), granulomatosis with polyangiitis (GPA), and eosinophilic granulomatosis with polyangiitis (EGPA) [1]. Renal involvement is common in patients with AGN and is closely associated with its prognosis [2–4]. The histologic hallmark of renal involvement in AGN is known as crescentic glomerulonephritis with fibrinoid necrosis. Though the molecular details of glomerular injury in AGN are still unclear, the interaction between ANCA and polymorphonuclear neutrophils, monocytes and macrophages might contribute to the glomerular injury in the kidney [5, 6].

Macrophages are part of innate immune system which are important to cytokine production and antigen presentation [7]. They could be differentiated from peripheral blood monocytes and be classified into M1 (classically-activated) subtype and M2 (alternatively activated) subtype [8]. It was demonstrated that ANCA could interact with macrophages by reducing their phagocytosis of apoptotic cells and thus lead to organ injury [9]**.** Further studies showed that M2 macrophages were associated with airways lesions in patients with GPA [10] and those cells were also positioned in the early stages of glomerular injury in patients with AGN [11]. All these results suggested M2 macrophages might play an important role in organ injury of AAV. CD206^+^ macrophage is a subtype of M2 macrophages which could interact with proinflammatory cytokines in autoimmune diseases and thus might be involved in the pathogenesis [12–16]. However, their role in AGN is not well studied. In this study, we investigate the roles of CD206^+^ mono-macrophages in AGN so as to provide insights of the pathogenesis of the disease.

## Method

### Patients recruitment and assessment

We recruited 27 AGN patients, including 14 newly diagnosed active AGN and 13 remissive AGN from Department of nephrology, Ruijin hospital affiliated to Shanghai Jiaotong University, School of medicine from January to December, 2021. AGN diagnosis was made when patients met the criteria of the Chapel Hill Consensus Conference definition for AGN [1] and positive for ANCA as we previously reported [17]. All the patients had renal involvement. Patients with secondary vasculitis including drug-induced vasculitis were excluded. Vasculitis disease activity was recorded using the Birmingham Vasculitis Activity Score (BVAS) 2003 [18]. Active AGN was defined as AGN patients with BVAS > 0 and remissive AGN were defined as AGN patients with BVAS = 0. Patients with persistent urinary abnormalities in the presence of improving or stable excretory kidney function, and no extrarenal disease activity, were determined to have a BVAS = 0 [19]. Among the patients with active AGN, renal biopsy was performed in 10 patients. The estimated GFR (eGFR) for active patients was calculated using the Chronic Kidney Disease Epidemiology Collaboration Creatinine Equation while considering the highest serum creatinine at diagnosis [20].The eGFR for remissive patients were calculated using Creatinine when they began their remission maintenance therapy. We collected laboratory data including ESR, CRP and leucocyte count as well. We also recruited 9 healthy controls, 6 disease controls and 9 kidney-function adjusted controls to make the comparison. Clinical data of kidney-function adjusted controls and disease controls were summarized in Additional file 1: Tables S1 and S3. Both disease controls and kidney-function adjusted controls were not on immunosuppressive therapy. The health controls were self-reported as healthy and had a normal urine test as well as normal kidney function. All the participants were above 18 yrs.

### Purification and enrichment of human MPO-ANCA IgG and control IgG

Human MPO-ANCA enriched IgG from active AGN patients and control IgG from healthy donors were purified and enriched by Protein G agarose purification kit (Sangon Biotech, Shanghai) according to manufacturer’s protocol. The purified antibody solution was detected by PAGE gel electrophoresis and Protein A280.The final solution was concentrated by ultrafiltration tube and centrifuged to 10 mg/ml, and then stored at − 80 °C for future use.

### Preparation of human peripheral blood mononuclear cells and macrophages

Under sterile conditions, anticoagulated whole blood was obtained from healthy donors through peripheral vein puncture, and further processing was carried out immediately. Density gradient centrifugation and hypotonic lysis of red blood cells were used to separate peripheral blood mononuclear cell (PBMC) to obtain high purity and viability. PBMCs were suspended in RPMI-1640 (Gibco) supplemented with 20 ng/ml Macrophage Colony Stimulating Factor (M-CSF, R&D Systems) to differentiate to PBMC derived macrophages. One week later, the cells were replaced with RPMI-1640 supplemented with 10% FBS (Gibco) and human MPO-ANCA enriched IgG or control IgG at a final concentration of 1 mg/ml, and then incubated at 37 °C for further study.

### Detection subtypes of monocytes in human peripheral blood by flow cytometry

Peripheral blood mononuclear cells were isolated from heparinized whole blood by Ficoll-Paque (GE Healthcare) density gradient centrifugation as standard protocol. All samples were stained with anti-CD68 antibody (BD Biosciences,), anti-iNOS antibody (BD Biosciences), anti-CD206 antibody (BD Biosciences) and anti-CD163 antibody (BD Biosciences). Stained cells were washed three times in flow cytometry buffer prior to flow cytometric analysis on BD LSRFortessa X-20 Flow Cytometer (BD Biosciences). Data were analyzed using FlowJo (BD Biosciences) software.

### Immunohistochemistry of CD206 in kidney tissues

Kidney tissues were sectioned and blocked with 3% H_2_O_2_. After heat retrieval and incubation with 1% BSA, sections were stained with antibodies specific for CD206 (Abcam) respectively followed by Horseradish peroxidase (HRP) labelled secondary antibodies (Jackson Immunoresearch) and HRP-labelled anti-Rabbit IgG (Jackson Immunoresearch). The slices were then stained with DAB and hematoxylin.

### Quantification of CD206 expression in kidney tissues

CD206 expression was quantified using the technique by the literature with slight amendment [11, 21]. Briefly, CD206 expression was scored according to the location of cells with each of four regions: (1) within regions of fibrinoid necrosis or crescent formation, (2) within regions of glomeruli, (3) in the periglomerular region, (4) within tubulointerstitial compartment. Scores were assigned as follows: Score 0: no CD206^+^ cells, Score 1: 1–5 CD206^+^ cells, Score 2: 6–10 CD206^+^ cells and Score 3: > 10 CD206^+^ cells. Values were calculated per glomerular cross section for each of the three glomerular regions, and by estimating the fraction of tubular or interstitial compartments.

### Measurement of soluble CD206(sCD206) in human serum samples

Serum sCD206 was detected by ELISA as standard procedure. Briefly, microtiter plates were coated with anti-CD206 antibody (R&D Systems). After blocking with PBS containing 1% BSA for 1 h at 37˚C, the microtiter plates were incubated with 50-fold diluted serum supernatants for 2 h at 37 °C, following adding in-house biotinylated monoclonal anti-CD206 antibody (Acris Antibodies) with incubation for 1 h at 37 °C, and HRP conjugated antibody (Jackson ImmunoResearch) for 1 h at 37 °C. The samples were analyzed spectrophotometrically at OD 450 nm.

### Mice

B6.129X1-*Mpo*^tm1Lus^/J (*Mpo*^−/−^) mice were purchased from The Jackson Laboratory (Stock No. 004265, Bar Harbor) and the DNA of tail clippings was obtained to identify genotype using polymerase chain reaction (PCR). This strain originated on a 129X1/SvJ background and has been backcrossed to C57BL/6 J for at least ten generations [22]. Wildtype C57BL/6 J (WT) mice were purchased from Vital River Laboratories (Shanghai, China), a joint venture of Charles River Laboratories. Mice were kept in the animal facility of Ruijin Hospital Affiliated to Shanghai Jiaotong University, School of medicine according to relevant regulations and all animal studies were approved by the Animal Care Committee of our institution.

### Preparation of murine myeloperoxidase (muMPO) ANCA

The *Mpo*^−/−^ mice was immunized as described with slight amendments [23]. Briefly, mouse MPO (muMPO, R&D System, Minneapolis) was mixed with Freund's adjuvant and injected intraperitoneally into *Mpo*^−/−^ mice. The muMPO was fully mixed with complete Freund's adjuvant during the first immunization on day 0, and then injected into *Mpo*^−/−^ mice intraperitoneally. The muMPO were mixed with incomplete Freund's adjuvant and injected for intensive immunization on the day 21, day 36, and day 42, respectively. Two weeks after day 42, mice were sacrificed, and the blood was collected.

### Detecting muMPO ANCA

muMPO ANCA were detected by enzyme-linked immunosorbent assay (ELISA) and indirect immunofluorescence (IF) in a standard protocol. For IF detection, neutrophils were incubated with mouse serum immunized with adjuvant solution with or without muMPO. Then, FITC-labeled fluorescent secondary antibody (Jackson ImmunoResearch) was incubated and observed under microscope. For ELISA, muMPO was used to coat the plate, and 100 μl ELISA coating solution containing 0.5 μg muMPO was added to each well following by blocking with PBS containing 1% BSA to reduce nonspecific binding. Mouse serum immunized with or without muMPO was diluted with PBS containing 1% BSA and incubated at 37 °C for 1 h. After incubation with HRP labeled secondary antibody (Jackson ImmunoResearch), TMB was added and the reaction was terminated by stopping solution. Finally, absorbance was measured at 450 nm wavelength.

### MPO activity assay

Murine MPO activity (muMPO) was detected by MPO activity assay kit (Abcam). Briefly, the samples were reacted with the reagents provided by the kit according to the manufacturer’s protocol. The samples were finally analyzed with a microplate reader at 412 nm.

### Preparation of mouse bone marrow-derived macrophages (BMDMs)

Mice were sacrificed, and femoral bones were obtained. Bone marrow cells (BMCs) from femoral bones were kept in growth medium for differentiation to macrophages. We used F4/80 as the marker for mouse macrophages to validate the differentiation of macrophages. To study effects of muMPO ANCA on CD206 and MPO expression in macrophages, BMDMs were cultured with RPMI1640 medium containing muMPO ANCA positive or negative mice serum for another 3 days. Then the cells were harvested for further study.

### Inhibiting MPO in BMDMs and PBMCs

To investigate effects of muMPO ANCA on CD206 expression, we used a specific MPO inhibitor AZD5904 (MedChemExpress, Shanghai) for cell intervention. BMDMs were kept in medium containing 10% muMPO ANCA positive mice serum treated with or without 20 μM AZD5904 and then harvested for further study. PBMCs were treated with human MPO-ANCA enriched IgG or control IgG at a final concentration of 1 mg/ml and was then incubated with or without 20 μM AZD5904 and harvested for further study. As CD206 is a glycosylated membrane protein, we extracted membrane protein of BMDMs using Mem-PER Plus membrane protein extraction Kit (Thermo Scientific) according to manufacturer’s protocol. The CD206 protein was then detected by western blot. We also performed immunofluorescence for CD206 staining in BMDMs to further validate the results of western blot.

### Western blotting

The procedures were performed according to a standard protocol. Briefly, the cell lysates were electrophoresed, blocked and then the PVDF membranes was incubated with anti-mouse CD206 (Abcam), Na–K ATPase (Abcam), β-actin antibody (Abcam) and anti-mouse MPO (Abcam) overnight at 4 °C. In some experiments, the membranes were cut prior to incubation with antibodies so as to minimize the amount of antibodies used in each experiment. Subsequently, the membranes were washed and incubated with HRP-conjugated secondary antibody (1:3000, Jackson immunoresearch). Proteins were finally visualized by ECL and ImageJ software (National Institute of Health) was used to observe band intensity.

### RT-PCR and real-time PCR

Total RNA was extracted from BMDMs with Trizol reagent (Invitrogen) following the manufacturer’s protocol. The reverse-transcription to cDNA was performed by a reverse transcription kit (Takara). Real-time PCR was performed using a standard SYBR Green PCR kit (Takara) to detect GAPDH, CD206, F4/80 and Arg-1 mRNA. Relative amounts of mRNA were normalized by GAPDH and calculated using the delta-delta method from threshold cycle numbers. Primer sequences were designed and synthesized by Biotech, Shanghai (Additional file 1: Table S2).

### Statistical analyses

Data were analyzed using SPSS 13.0 (SPSS Inc.). Data with normal distribution were summarized as mean ± SD. Data without normal distribution were summarized as median and interquartile range. Differences between two groups were compared by unpaired Student’s *t*-tests or Mann–Whitney *U* tests and differences among multiple groups were performed using one-way analysis of variance (ANOVA). Data were considered statistically significant if *p* < 0.05.

## Results

### Clinical characteristic of patients

In our study, there were 14 MPA patients in the active vasculitis group; 1 RLV,1 GPA and 11 MPA patients in the remissive vasculitis group. The clinical and laboratory features of the AGN patients were summarized in Table 1.

**Table 1:** Clinical and laboratory presentation of *AGN* patients

Characteristics	Patients with active AGN (n = 14)	Patients with remissive AGN (n = 13)
Sex (male, %)	10 (71.4)	4 (30.8)
Age (yrs, mean)	68.9	66
MPO-ANCA positivity/PR3-ANCA positivity	13/1	12/1
Extrarenal involvement (n, %)		
Lung and upper respiratory tract	12 (85.7)	–
ENT	6 (42.9)	–
Nervous system	2 (14.3)	–
Cutaneous/mucous membranes/eyes	2 (14.3)	–
Fever	5 (35.7)	–
BVAS (median, range)	20.5 (12–29)	–
Renal involvement		
Proteinuria (mg/24 h, median [range])	1653 (276–5242)	560 (87–1249)
Serum creatinine (μmol/L, mean ± SD)	590.9 ± 105.9	209.5 ± 33.4
eGFR (ml/min, mean ± SD)	12.4 ± 2.4	32.0 ± 5.2
Laboratory perimeters		
ESR(mm/h, mean ± SD)	55.9 ± 38.8	10.1 ± 7.5
CRP(mg/L, mean ± SD)	43.2 ± 60.5	1.0 ± 0.6
White blood cell count (× 10^9^/L, mean ± SD)	9.3 ± 3.6	8.2 ± 2.8

ENT, ear, nose, and throat; eGFR, estimated glomerular filtration rate; ESR, erythrocyte sedimentation rate; CRP, C-reactive protein

### CD206^+^CD68^+^ monocytes were increased in peripheral blood of patients with AGN

To study the subtypes of monocytes in peripheral blood of patients of AGN, we used iNOS^+^CD68^+^, CD206^+^CD68^+^ and CD163^+^CD68^+^ as the markers for the cells.

**Fig. 1:**
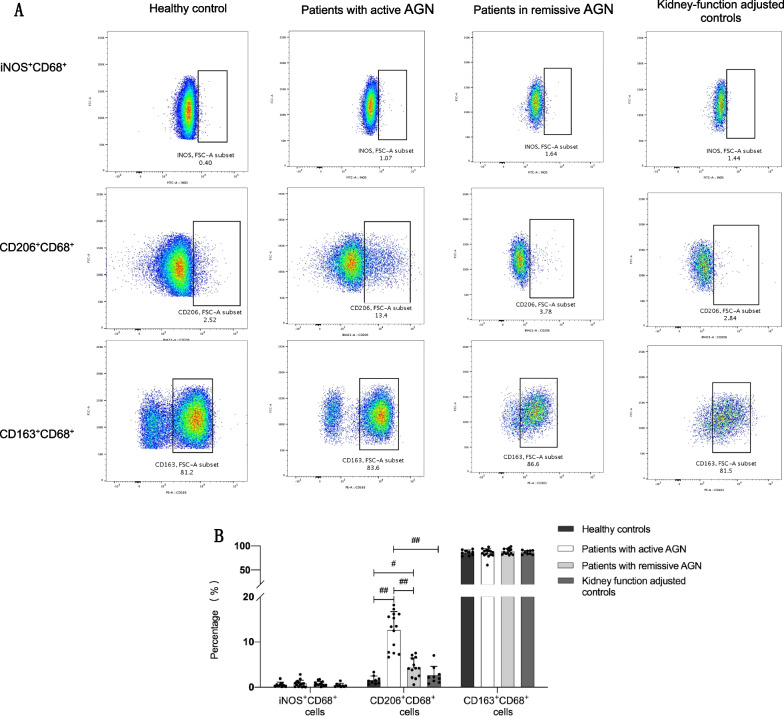
Subtypes of peripheral monocytes in patients with AGN. Peripheral blood was collected from healthy controls (n = 9), kidney-function adjusted controls (n = 9), patients with active AGN (n = 14) and patients with remissive AGN (n = 13). Samples were analyzed by flow cytometry to investigate proportion of different subtypes of cells including iNOS^+^CD68^+^, CD206^+^CD68^+^ and CD163^+^CD68^+^ cells. **A** A representative flow cytometry analysis of iNOS^+^CD68^+^ cells, CD206^+^CD68^+^ cells and CD163^+^CD68^+^ cells in peripheral blood of one individual from each group. **B** Histograms showed the proportions of different cells from each group. Dots represented for the number of proportions of iNOS^+^CD68^+^, CD206^+^CD68^+^ and CD163^+^CD68^+^ cells from each group and error bars represented for the standard deviation (SD). Compared with other groups, patients with active AGN presented with high proportion of CD206^+^CD68^+^ cells (*p* < 0.001). Patients with remissive AGN had higher proportion of CD206^+^CD68^+^ cells in comparison with healthy controls with statistical significance (*p* < 0.01). #*p* < 0.01; ##*p* < 0.001

Our results showed that the proportion of CD206^+^CD68^+^ cells in patients with active AGN were significantly higher than that in patients in remissive AGN (12.62 ± 4.13% vs 4.29 ± 2.17%, *p* < 0.001), healthy controls (12.62 ± 4.13% vs 1.56 ± 0.88%, *p* < 0.001) and kidney-function adjusted controls (12.62 ± 4.13% vs 2.63 ± 1.94%, *p* < 0.001). Compared with that in healthy controls, the proportion of CD206^+^CD68^+^ cells in patients with remissive AGN increased significantly (4.29 ± 2.17% vs 1.56 ± 0.88%, *p* < 0.01) (Fig. 1). There was no significant difference regarding proportion of CD206^+^CD68^+^ cells between remissive AGN patients and kidney-function adjusted controls (*p* > 0.05). The proportion of CD163^+^CD68^+^ cells and iNOS^+^CD68^+^ cells did not change significantly between different groups. The flow cytometry results suggested the CD206^+^ monocytes were activated in patients with AGN and might be associated with disease activity.

### CD206 was highly expressed in kidney tissues of patients with active AGN

As circulating monocytes cells could be recruited and positioned in the kidney to differentiate to macrophages, we then analyzed the expression of CD206 in kidney tissues of patients. Kidney biopsy were available in 10 patients with active AGN, we also recruited 6 disease controls with kidney biopsies.

**Fig. 2:**
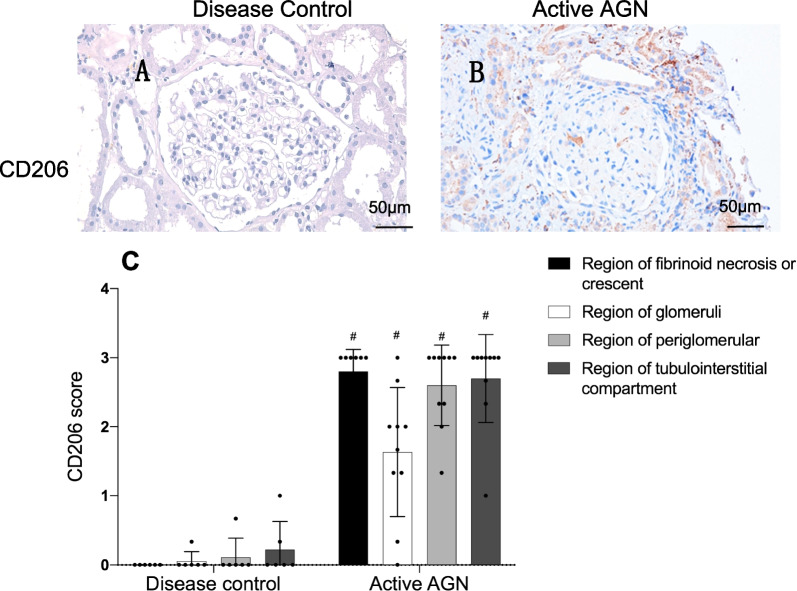
CD206 staining in patients with active AGN. **A**, **B** Images depict representative high-power views of paraffin-embedded human kidney sections of disease control (**A**) and active vasculitis (**B**). CD206 were stained by immunohistochemistry. No positive staining for CD206 were found in the disease control (**A**). **C** Histograms of CD206 staining areas in the glomeruli from patients with AGN (n = 10) and disease control (n = 6). Paraffin-embedded human kidney sections from patients with vasculitis were stained for CD206 protein by immunohistochemistry and scored blind according to the location of cells with each of four regions: (1) within regions of fibrinoid necrosis or crescent formation, (2) within regions of glomeruli, (3) in the periglomerular region, (4) within tubulointerstitial compartment. Dots represented for the scores from different regions and error bars represented for the standard deviation (SD). The results showed that compared with disease controls, CD206 scores were significant higher in regions of fibrinoid necrosis or crescent formation (2.80 ± 0.32 vs 0); periglomerular region (1.63 ± 0.94 vs 0.06 ± 0.14), tubulointerstitial compartment (2.60 ± 0.58 vs 0.11 ± 0.27) and glomeruli (2.70 ± 0.64 vs 0.22 ± 0.40) in AGN patients. #*p* < 0.01

By using immunochemistry for CD206, our results showed that CD206 was highly expressed in patients with AGN (Fig. 2). We did not find positive staining for CD206 in disease controls. We used CD206 score for quantification of CD206 expression in the kidney tissues. Our results showed that CD206 expression was prominent in glomerular injury as well as tubulointerstitium in AGN patients.

### Serum sCD206 was elevated in patients with active AGN

CD206 is a membrane protein which could be cleaved to form soluble CD206 (sCD206), we then examined the serum sCD206 levels with disease activity in patients with AGN.

**Fig. 3:**
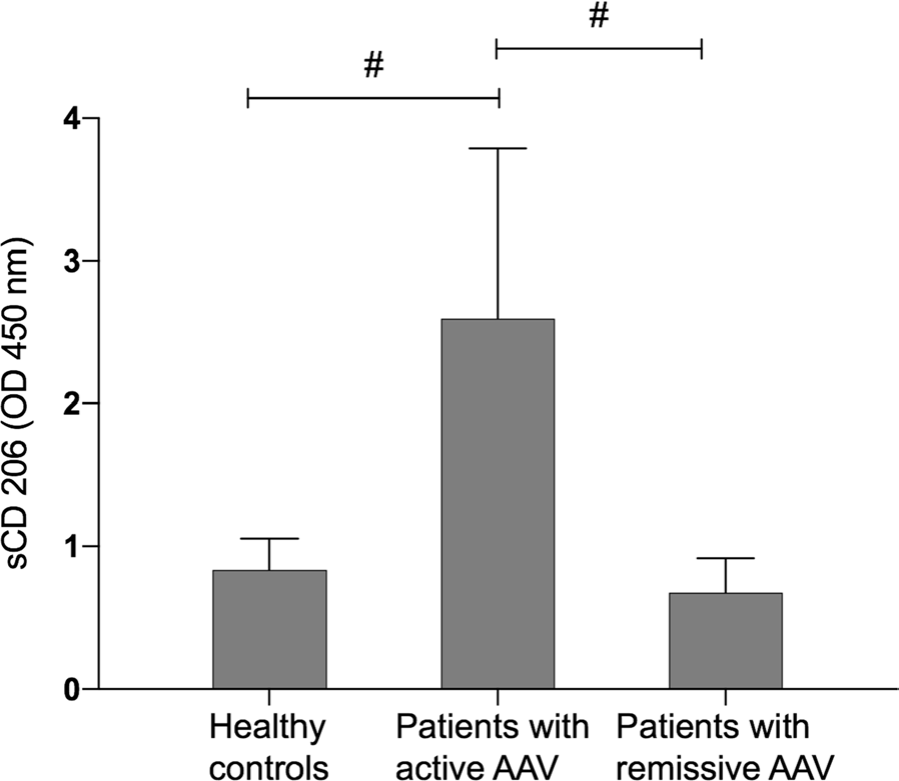
Serum sCD206 levels in patients with AGN. Serum sCD206 levels from healthy controls (n = 9), patients with active AGN (n = 14) and patients with remissive AGN (n = 13) were detected by value of ELISA in absorbance at OD 450 nm.Compared with data in healthy controls and remissive AGN patients, sCD206 levels increased with statistical difference (*p* < 0.01). There was no statistical difference regarding sCD206 levels between healthy controls and remissive AGN patients. Dots represented for the exact number of ELISA value from different groups and error bars represented for the standard deviation (SD). #*p* < 0.01

Our results showed that compared with healthy controls, level of serum sCD206 in patients with active AGN increased significantly (0.83 ± 0.22 vs 2.60 ± 1.19, *p* < 0.01). Meanwhile, level of serum sCD206 in active AGN group increased significantly (0.67 ± 0.24 vs 2.60 ± 1.19, *p* < 0.01) in comparison with that in remissive AGN group. There was no statistical significance regarding level of serum sCD206 between healthy controls and patients with remissive AGN (*p* > 0.05) (Fig. 3).

### MPO-ANCA could induce CD206 expression in both mice bone-marrow derived macrophages as well as human PBMC derived macrophages and such effects could be reversed by MPO inhibitor

**Fig. 4:**
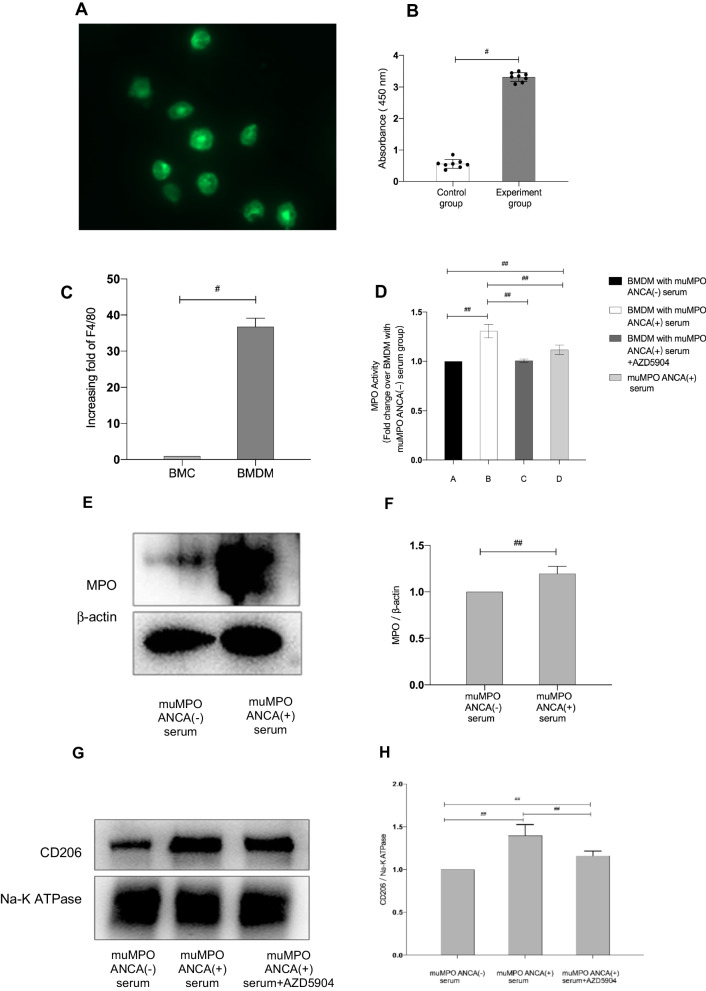

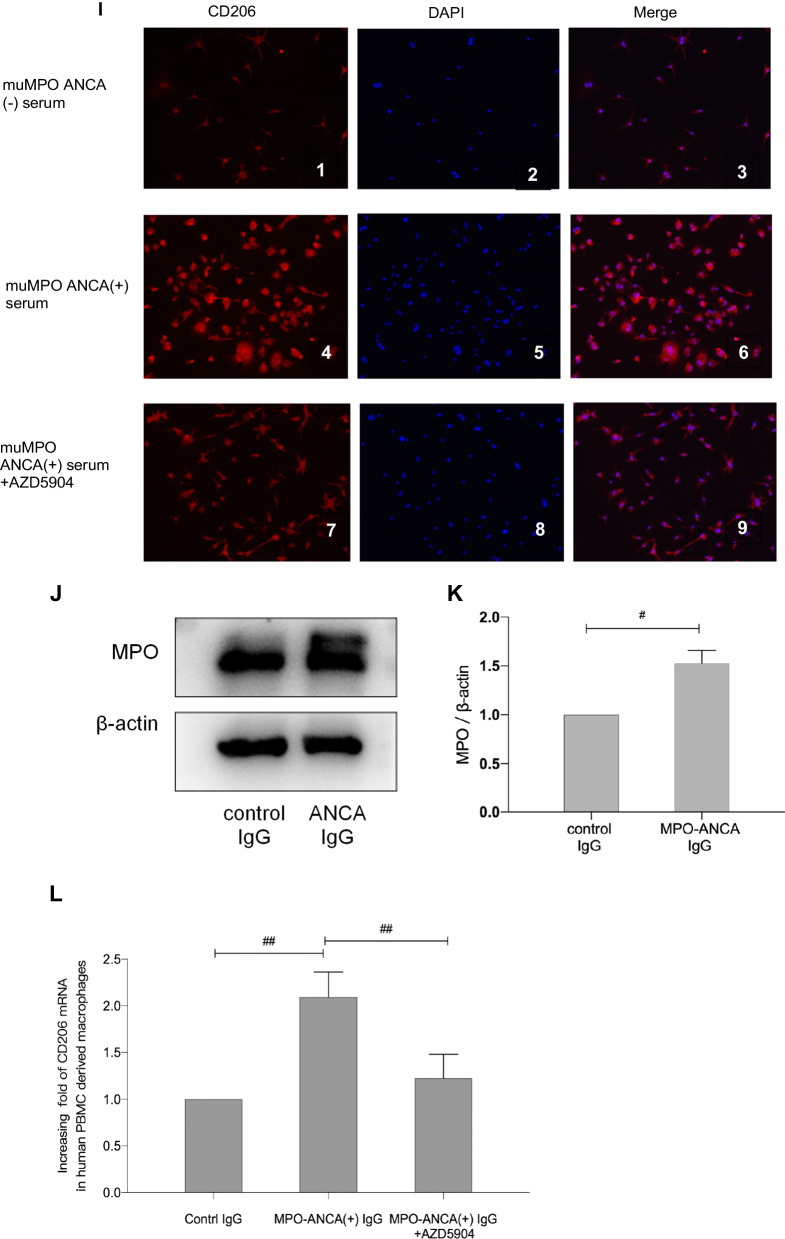
Effects of MPO-ANCA on CD206 expression in macrophages. **A** The indirect immunofluorescence (IF) showed that a representative of *Mpo*^−/−^ mice immunized with muMPO developed muMPO ANCA in neutrophils. **B** The presence of muMPO ANCA was determined by ELISA. The results showed that compared with control mice (n = 8), muMPO ANCA were positive in the immunized mice (n = 8). **C** Mouse bone marrow cells (BMCs) differentiated to BMDMs and total mRNA was extracted for validation of macrophages differentiation. We used F4/80 as the marker for mouse macrophages. The real-time PCR result showed that compared with untreated group, the expression of F4/80 in BMDMs increased with statistical significance (*p* < 0.001). The results confirmed the successful differentiation of bone marrow derived macrophages. **D** The MPO activity assay showed that MPO activity in BMDMs with muMPO ANCA positive serum group increased significantly in comparison with that in BMDMs with control serum (*p* < 0.05) or muMPO ANCA positive serum without BMDMs (*p* < 0.05). Such effects could be reversed by MPO inhibitor AZD5904 (*p* < 0.05). **E**, **F** BMDMs were treated with muMPO ANCA positive serum or control serum. The expression of MPO in BMDMs were determined by western blot (**E**) and analyzed by densitometry (**F**). Western blot showed the results from one of three independent preparations. The results were showed in cropped blots and densitometrical analysis were results from three independent cell preparations. **G**–**I** BMDMs were treated with serums from different groups of mice. The expression of CD206 was determined by western blot (**G**) and immunofluorescence (**I**). For the western blot, the membrane proteins of BMDMs were extracted and Na–K ATPase was used to verify equivalent loading (**G**) and shown in cropped blots. For the immunofluorescence, the slides of cells were stained for CD206 and DAPI (**I**). Densitometrical analysis (**H**) results shown were results from three independent cell preparations. Immunofluorescence and western blot showed the results from one of three independent preparations. **J**, **K** PBMC derived macrophages from healthy donors were treated with control IgG or MPO-ANCA enriched IgG. The expression of MPO were determined by western blot (**J**) and analyzed by densitometry (**K**). Western blot showed the results from one of three independent preparations. The results were showed in cropped blots and densitometrical analysis were results from three independent cell preparations. **L** PBMC derived macrophages from healthy donors were treated with control IgG or MPO-ANCA enriched IgG with or without AZD5904. The real-time PCR result showed that CD206 mRNA was significantly increased in the presence of MPO-ANCA enriched IgG and such effects could be counteracted by the presence of AZD5904. #*p* < 0.001, ##*p* < 0.05

To further study the effects of ANCA on CD206 expression, we generated muMPO ANCA by immunizing *Mpo*^−/−^ mice. As shown in Fig. 4A–C, the immunized *Mpo*^−/−^ mice developed muMPO ANCA which were confirmed by indirect IF and ELISA.

We firstly used mouse bone marrow cells (BMCs) to differentiate to bone marrow derived macrophages (BMDMs) to study the MPO activity in the supernatant of BMDMs. Our results (Fig. 4D) showed that compared with controls, BMDMs incubated with muMPO ANCA had significantly increased MPO activity in the supernatant (*p* < 0.05). Subsequently, we performed western blot to detect MPO expression in BMDMs. As shown in Fig. 4E, F, MPO expression in BMDMs treated with muMPO ANCA increased significantly in comparison with controls. Our results thus demonstrated that muMPO ANCA could enhance expression of MPO in BMDMs.

We then investigate effects of MPO-ANCA on CD206 expression. By using western blot for membrane protein and immunofluorescence, our results showed that BMDMs treated with muMPO ANCA had higher level of CD206 expression in comparison with the those treated with control (*p* < 0.05). Inhibiting MPO by AZD5904 could significantly reduce CD206 expression (Fig. 4G–I).

Lastly, we used PBMC derived macrophages from health donors to validate our results. Similar to the results in BMDMs, MPO-ANCA enriched IgG from AGN patients could significantly induce the expression of MPO in PBMC derived macrophages (Fig. 4J, K). By using real-time PCR, our results further showed that MPO-ANCA enriched IgG could significantly increase the expression of CD206 mRNA in PBMC derived macrophages (*p* < 0.001) and such effects could be reversed by MPO activity inhibitor AZD5904 (*p* < 0.05) (Fig. 4L). Our findings thus suggested MPO-ANCA could induce CD206 expression in macrophages and such effects could be reversed by inhibiting enzymatic MPO activity.

## Discussion

ANCAs are traditionally known to interact with neutrophils to cause respiratory burst and organ damage [24–26]. However, studies point out that monocytes and macrophages are also important to tissue and blood vessels injury during AGN [27]. Several studies [11, 21, 28] including our current study demonstrated macrophages and monocytes were important to organ damage of AGN, thus the role of these cells in organ damage requires intensive investigation.

In current study, we found high proportion of CD206^+^ monocytes in peripheral blood of patients with active AGN. These activated monocytes would then differentiate to CD206^+^ macrophages which is a subtype of alternatively activated macrophages that express proinflammatory cytokines and play important roles in many kidney diseases and autoimmune disorders [15]. Li and colleagues showed in their study that number of CD206^+^ cells were negatively correlated with eGFR in patients with acute tubulointerstitial injury [29]. In the study focusing on patients with IgA nephropathy, Xie and colleagues found that CD206^+^ and CD68^+^ macrophages infiltrated in the glomeruli were correlated to treatment response of immunosuppressive therapy [30]. Similar results were also found by Hu et al. who demonstrated that CD206^+^ macrophages were correlated with the clinical features in patients with IgA nephropathy [31]. In further study of macrophages in the autoimmune diseases, Deng and colleagues [16] reported that promoting CD206 expression of macrophages could alleviate systemic involvement of lupus by inducing phagocytic activity. Harper et al. [9] showed in their study that incubating neutrophils with ANCA and ANCA-induced apoptotic neutrophils could not be cleared up by murine peritoneal-derived macrophages. They speculated that ANCAs could accelerate apoptosis and secondary necrosis which was related to “reduced window of opportunity” for phagocytic recognition and engulfment before disintegration. Nevertheless, role of CD206^+^ macrophages in AGN is not well studied. In present study, we demonstrated that CD206^+^ macrophages infiltrated in the glomerular of patients with AGN. Considering high proportion of CD206^+^ monocytes in active AGN patients and circulating monocytes could be recruited and differentiated to macrophages in the kidney and orchestrate immune response, our results imply that CD206^+^ mono-macrophages might be involved in the pathogenies of AGN and organ damage, but further studies are necessary to study the molecular details during this process.

CD206 (also termed as mannose receptor, MR) is a glycosylated membrane protein which belongs to a group of scavenger receptors expressed by macrophages, monocytes and other cells [32, 33]. It contains a cysteine-rich region, a fibronectin type II domain, eight C-type lectin-like domains, a transmembrane region and a short tail [34]. By binding to a series of ligands and promoting removal of foreign substances or changing self-substances, CD206 plays an important role in antigen uptake and presentation [13, 35]. In our study, we found muMPO ANCA could induce CD206 expression in BMDMs and such effect could be reversed by MPO inhibitor-AZD5904. We searched the literatures and found that MPO-ANCA could reduce the expression of interleukin (IL)-6 and IL-10 in response to toll-like receptor 4 (TLR4) stimulation in monocytes, which depended on MPO enzymatic activity through an MPO-driven increase in oxidized phospholipids pathway [36]. MPO can catalyze oxidation reactions and produce a variety of oxidized lipids which could trigger inflammatory response and regulate gene expression as well as cellular metabolism in macrophages [37–39]. In our study, MPO-ANCA could induce CD206 expression in macrophages and such effects could be reversed by AZD5904. Consider the interacting between MPO and oxidized phospholipids, we hypothesize that oxidized phospholipids might be involved in AGN and AZD5904 might affect the production of oxidized phospholipids. However, more studies are necessary to investigate this process.

It is known that CD206 could be cleaved by metalloprotease to produce a soluble form-sCD206, which is present in plasma [40]. sCD206 is considered a macrophage activation marker and might be related to disease activity including sepsis, liver disorders and autoimmunity [41–44]. In our study, we found serum sCD206 levels elevated in active AGN patients and decreased in remissive ones. As it was also shown in our study that CD206^+^ mono-macrophages were more prominent in patients with active AGN, serum sCD206 could thus be a potential marker for disease. Moreover, studies show that sCD206 is important to cytokine production and macrophage polarization. In the study by Embgenbroich and colleagues [34], sCD206 directly interacted with CD45 on the surface of macrophages, leading to Src/Akt/NF-κB-mediated cellular reprogramming toward an inflammatory phenotype. Klotz et al. [45] found increased sCD206 levels could be related to peroxisome proliferators-activated receptors (PPAR)-γ activation which could trigger downstream cytokine production. Furthermore, it was shown that MPO-ANCA could increase monocyte survival and differentiating to macrophages by interacting with proinflammatory cytokines like IL-6 and IL-10 [36]. Since macrophage could secrete sCD206, MPO-ANCA might thus contribute to increased level of sCD206, but the molecular details during this process requires further studies.

Meanwhile, we searched the literature and found soluble CD163 (sCD163), another soluble membrane protein of macrophage, expressed differently in the serum of patients with AGN. Different to the expression of urinary sCD163 which was elevated in active and relapsed AGN patients [46], serum sCD163 did not correlate with disease activity [21]. It was found that the infiltration of CD163^+^ macrophages in AAV kidney increased in accordance with the increased level of urinary sCD163. However, no significant elevation of sCD163 was found in the serum of AAV patients in comparison with that in the controls. It was hypothesized that the increased CD163^+^ macrophages in the kidney were recruited from local region rather than peripheral blood [47]. Different from CD163, sCD206 production may be constitutive and the plasma concentration of sCD206 is increased in a wide range of diseases [43, 48–50]. Though CD206 and CD163 belong to similar membrane protein of macrophage, different profiles of sCD206 and sCD163 suggested that they might play different roles in the pathogenesis of AGN.

There are some limitations in our study we must acknowledge. First, the number of patients in the kidney-function adjusted controls and disease-controls were small. Second, the retrospective nature of data collection and the disease heterogeneity of AGN prevented us from getting further information. Last, we did not examine urinary sCD206 in our patients. As some of our patients with active vasculitis presented with oliguria at disease onset, their urine samples were not available. Therefore, a large cohort is necessary to study sCD206 in both serum and urine samples of patients to study their correlation with disease activity.

## Conclusion

Our study demonstrates that ANCA could induce CD206 expression and CD206 might be involved in the pathogenesis of AAV. CD206^+^ mono-macrophages are activated in patients with AGN and are involved in kidney injury. Our findings thus suggest these macrophages and sCD206 could be a potential target for patients with AGN.

## Supplementary Information

**Additional file 1: Table S1**. Clinical and laboratory presentation of kidney-function adjusted controls. **Table S2**. PCR primer sequence. **Table S3**. Clinical and laboratory presentation of disease controls. **Figure S1**. The original images of Fig. 4E. **Figure S2**. The original images of Fig. 4G. **Figure S3**. The original picture of Fig. 4J.

## Data Availability

All data that support the findings of this study are available from the corresponding author upon reasonable request.

## Abbreviations

ANCA: Antineutrophil Cytoplasmic Antibodies
AGN: Antineutrophil Cytoplasmic Antibodies associated glomerulonephritis
BMCs: Bone marrow cells
BMDMs: Bone marrow-derived macrophages
PBMC: Peripheral blood mononuclear cell
MPA: Microscopic polyangiitis
GPA: Granulomatosis with polyangiitis
EGPA: Eosinophilic granulomatosis with polyangiitis
BVAS: Birmingham Vasculitis Activity Score
M-CSF: Macrophage Colony Stimulating Factor
sCD206: Soluble CD206
