# Supplementary material for: CD206+CD68+ mono-macrophages and serum soluble CD206 level are increased in antineutrophil cytoplasmic antibodies associated glomerulonephritis
Source: BMC Immunol. 2022 Nov 15;23:55. doi: 10.1186/s12865-022-00529-w (PMC9664714; doi:10.1186/s12865-022-00529-w)

**Additional file 1: Table S1**. Clinical and laboratory presentation of kidney-function adjusted controls

|  | kidney-function adjusted controls (*n*=9) |
| --- | --- |
| Gender (Male, %) | 7(77.8) |
| Age (yrs, mean) | 49.2 |
| Proteinuria (mg/24h, median [range]) | 2768(203-9057) |
| Serum Creatinine (μmol/L, mean±SD) | 437.8±283.0 |
| eGFR (ml/min, mean±SD) | 20.9±18.2 |
| Diagnosis (n, %) |  |
| Diabetes nephropathy | 1(9) |
| IgA nephropathy | 2(18.1) |
| primary glomerulonephritis | 6(66.7) |

eGFR: estimated glomerular filtration rate

**Additional file 1: Table S2.** PCR primer sequence

| Gene | Primer sequence(forward) | Primer sequence(reverse) |
| --- | --- | --- |
| GAPDH (mouse) | 5’- AGAAGGTGGTGAAGCAGGCATC-3’ | 5’-CGGCATCGAAGGTGGAAGAGTG-3’ |
| F4/80 (mouse) | 5’-TGTCTGCATGATCATCACGATA-3’ | 5’-CGTGTCCTTGAGTTTAGAGACT-3’ |
| GAPDH (human) | 5’- AAGGTCGGAGTCAACGGATTT-3’ | 5’- AGATGATGACCCTTTTGGCTC-3’ |
| CD206 (human) | 5’- ACACAAACTGGGGGAAAGGTT-3’ | 5’- TCAAGGAAGGGTCGGATCG-3’ |

**Additional file 1: Table S3**. Clinical and laboratory presentation of disease controls

|  | disease controls (n=6) |
| --- | --- |
| Sex (Male, %) | 4(66.7) |
| Age (yrs, mean) | 36 |
| Proteinuria (mg/24h, median [range]) | 6507.5(1420-17387) |
| Serum Creatinine (μmol/L, mean±SD) | 183.2±165.1 |
| eGFR (ml/min, mean±SD) | 77.7±63.9 |
| Pathological diagnosis (n,%) |  |
| Focal segmental glomerulosclerosis | 3(50.0) |
| Minimal change disease | 2(33.3) |
| IgA nephropathy | 1(16.7) |

eGFR: estimated glomerular filtration rate

**Additional file 1: Figure S1.** The original images of Figure 4E

β-actin No.1 MPO No.1


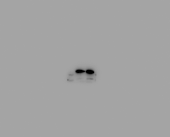

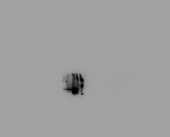


β-actin No.2 MPO No.2


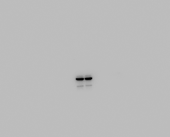

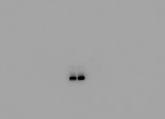


β-actin No.3 MPO No.3


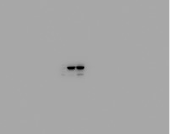

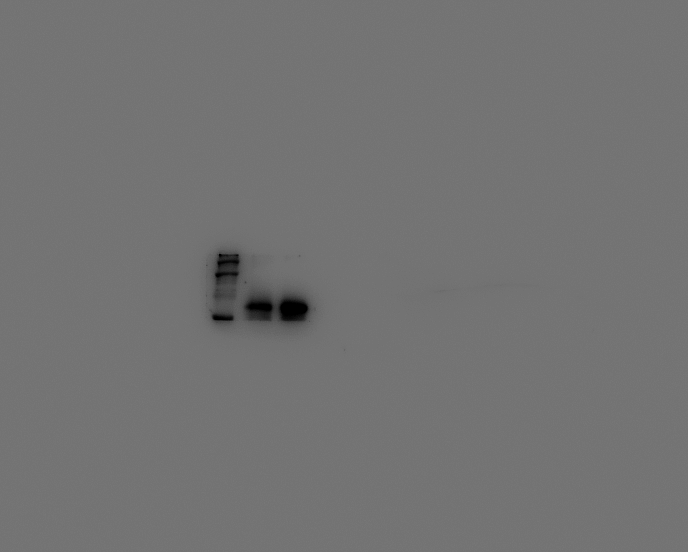


**Additional file 1: Figure S2:** The original images of Figure 4G

Na-K ATPase No.1 CD206 No.1


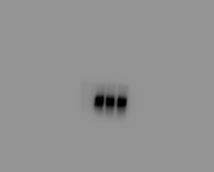

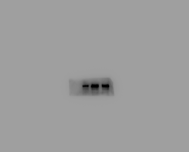


Na-K ATPase No.2 CD206 No.2


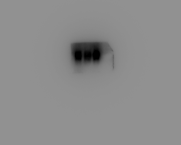

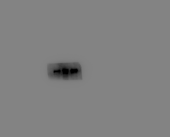


Na-K ATPase No.3 CD206 No.3


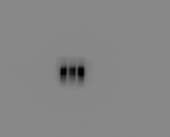

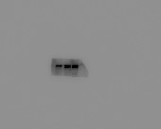


**Additional file 1: Figure S3.** The original picture of Figure 4J

β-actin


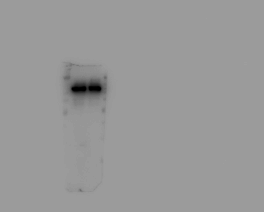


MPO


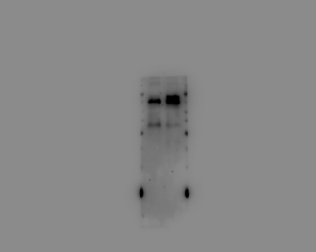

Supplement: Supplementary file 1 — Additional file 1: Table S1. Clinical and laboratory presentation of kidney-function adjusted controls. Table S2. PCR primer sequence. Table S3. Clinical and laboratory presentation of disease controls. Figure S1. The original images of Fig. 4E. Figure S2. The original images of Fig. 4G. Figure S3. The original picture of Fig. 4J. [file 12865_2022_529_MOESM1_ESM.docx]
